# Experimental Study on Double-Joint Soft Actuator and Its Dexterous Hand

**DOI:** 10.3390/mi14101966

**Published:** 2023-10-22

**Authors:** Bingxing Chen, Qiuxu Meng, Junjie Wang, Zongxing Lu, Yingjie Cai

**Affiliations:** School of Mechanical Engineering and Automation, Fuzhou University, Fuzhou 350108, China; bingxingchen@fzu.edu.cn (B.C.); 230227053@fzu.edu.cn (Q.M.); wjj675112@gmail.com (J.W.); cyj@fzu.edu.cn (Y.C.)

**Keywords:** double-joint soft actuator, bend performance, dexterous robotic hand

## Abstract

In this paper, we propose a pneumatic double-joint soft actuator based on fiber winding and build a dexterous hand with 11 degrees of freedom. Firstly, soft actuator structural design is carried out according to the actuator driving principle and gives the specific manufacturing process. Then, an experimental analysis of the bending performance of a single soft actuator, including bending angle, speed, and force magnitude, is carried out by building a pneumatic control experimental platform. Finally, a series of dexterous robotic hand-grasping experiments is conducted. Different grasping methods are used to catch the objects and measure the objects’ change in height, length, and rotation angle during the experiment. The results show that the proposed soft actuator is more consistent with the bending rule of human fingers, and that the gestures of the dexterous hand are more imaginable and flexible when grasping objects. The soft actuator can carry out horizontal and vertical movements, and rotation of the object in the dexterous hand, thus achieving better human–computer interaction.

## 1. Introduction

Traditional robots are mostly made of non-deformable rigid materials, while manipulators, as indispensable end-effectors of robots, constantly appear in various production activities or complex environments, playing an essential role in accelerating industrial production and executing complex, changeable, or dangerous grasping requirements [1]. However, due to the complex structure of rigid manipulators, their difficulty to control, their relatively high production cost, and their lack of certain flexibility and safety when grasping objects due to fixed contact, the application and development of manipulators are limited [2,3].

Scientists have been inspired by natural creatures such as octopus tentacles [4], plant twines [5], spiders [6], and fish [7] to create soft robots that mimic humans. In the rehabilitation training of people with hand injuries and the development of rehabilitative hands [8,9], surgical treatment [10], and diving operations [11,12], soft robots show higher safety and flexibility, and better human–machine interaction. Flexible manipulators composed of soft actuators have achieved remarkable results because of their soft contact material and simple structure, showing high safety and flexibility when grasping objects. Their design concept can be extended to flexible hands with strong grip, precision grip, and dexterous hand manipulation [13]. Some flexible manipulators can also be made via modular construction, so rapid prototyping is performed to achieve different functions [14]. From a three-finger soft grip to a five-finger soft grip, a dexterous hand offers an excellent gripping or grasping effect on objects [13,14,15,16]. Scientists have improved the gripping capability of actuators by combining multiple actuation methods [17,18,19], increasing the material filling, using fiber material composition [20,21], and adopting structural optimization methods to enhance the rigidity of actuators [22,23,24,25,26]. However, when limited by a single degree of freedom, actuators perform one-piece bending. They cannot effectively simulate human finger joint independent bending, which leads to a limited contact area with the object when grasping, and dexterous hands with relatively few degrees of freedom of control, hindering the reliability of grasping. It is difficult to achieve in-hand horizontal and vertical movements and the rotating of objects by dexterous hands, thus greatly limiting application and development.

In this paper, according to the structural characteristics of human fingers and the principle of actuator bending, we designed a bionic soft actuator with two degrees of freedom, which is very close to the bending law of the human finger. The manufacturing process is then described. Section 2 outlines our analysis of the bending performance of a soft actuator, which we conducted by building an experimental platform with an air pump that can adjust gas flow rate, thus enabling a change in the speed of actuator bending during the gripping process. Section 3 describes the process of building a five-finger dexterous hand with 11 degrees of freedom based on the designed soft actuator. Five fingers adopt the same structure with a total of 10 degrees of freedom, and an additional degree of freedom is added by setting a steering engine at the root of the thumb. The feasibility of the actuator is analyzed. It is proven that the dexterous hand made using the proposed actuator has better human–computer interaction performance because of its excellent bending performance. The proposed dexterous hand can realize in-hand manipulation, including horizontal and vertical movements and rotation of the object. At the same time, the utilization of flexible materials in the composition of the entire dexterous hand significantly simplifies both its structure and manufacturing cost.

## 2. Structural Design Fabrication

### 2.1. Soft Actuator Structure Design

**Figure 1:**
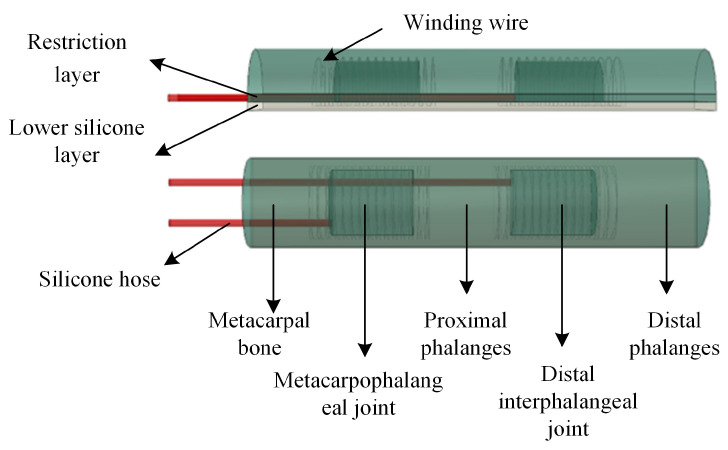
Soft actuator structure.

The soft actuator is based on the pneumatic fiber-reinforced drive principle, and the central part is made of silica gel material. The soft actuator (Figure 1) is mainly divided into a silicone gas pipe, an axial fiber restraint layer, an upper and lower silicone body, and a winding thread. The silicone body consists of two flexible joints, two finger bones, and the lower part of the metacarpal, which acts as the fixing end. In the actuator bending experiment, gas can be passed into the corresponding joint through two silicone gas tubes. Because the radial winding line of the joint restricts the expansion of the actuator joint, the limiting layer at the bottom restricts the axial stretch of the actuator, resulting in the bending deformation of the actuator in the joint part. At the same time, the two joints are entirely independent of each other, which can achieve the effect of different degrees of bending angles of the two joints. Because finger bones are made of solid silica gel, they do not bend. The overall bending angle of the actuator can be regarded as the superposition of the bending angles of two air cavities, which conforms to the structural characteristics of human fingers and can effectively fit the surface of the grasped object.

### 2.2. Soft Actuator Simulation Model and Parameter Determination

**Figure 2:**
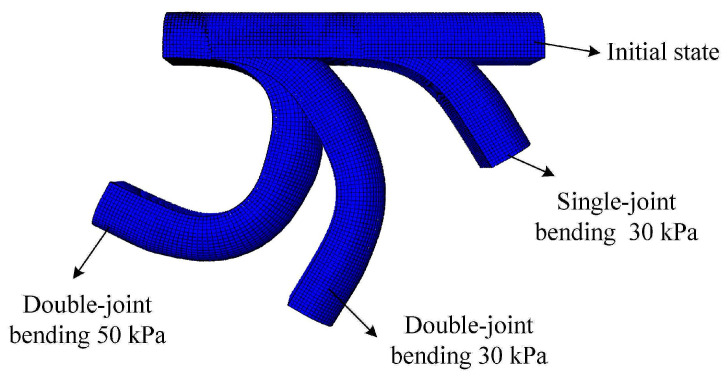
Bending deformation diagram of the soft actuator.

After determining the deformation mechanism and the actuator’s structure, finite element simulation software abacus was used to analyze the actuator to find the correct structural parameters of the soft actuator. The mesh property of the silica gel material was set to an eight-node linear hexahedron element, C3D8H; the axial fiber limiting layer was set to a four-node curved thin shell, S4R; the winding part was set to a secondary beam element, B32; and the interaction part was set to the built-in constraint modeling the actuator. Internal pressure perpendicular to the surface was applied to the inside of the actuator. The bending deformation effect of the actuator is shown in Figure 2, without considering the influence of gravity on the actuator.

**Figure 3:**
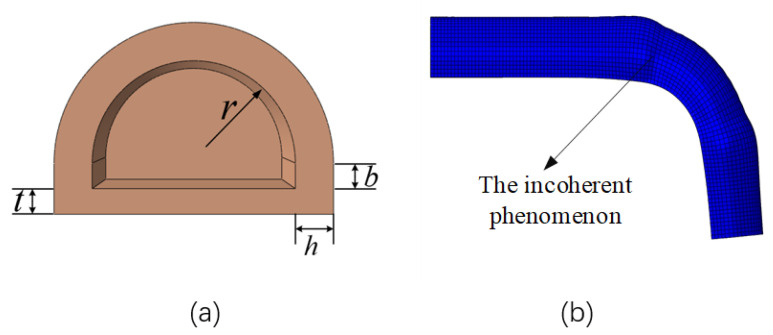
(**a**) Soft actuator cross-section shape. (**b**) Simulation incoherent effect.

In an early experiment, the influences of structural parameters such as joint length, wall thickness, radius, coil number, and cavity section rectangle height on the bending performance of the actuator were studied to determine the optimal structural parameters of the actuator. Based on the semicircle section [27], the semicircle plus rectangle section shape was determined to be more conducive to the bending and deformation of the actuator. The section shape diagram and structural parameters of the actuator are shown in Figure 3a and Table 1. In the simulation experiment of the number of winding coils of the actuator, to better realize the bending effect of the actuator and to prevent the separation of the upper and lower layers of the actuator, the incoherent phenomenon occurred in the joint and finger bone when the actuator was bent, as shown in Figure 3b. At the same time, considering that the length of the air cavity of the actuator was finally determined to be 20 mm, the winding distance was expanded to 30 mm, the number of winding coils was set to 16 turns, and the corresponding helical pitch was set to 2 mm. The reasonable selection of the number of winding coils limits the circumferential expansion of the actuator, which is conducive to enhancing the bending effect of the actuator [28]. We selected a small segment of actuators as our study objects. The actuator segment length, $u_{0}$, and width, $v_{0}$, can be expressed as follows:

(1) $$u_{0}=\frac{H}{2n}$$

(2) $$v_{0}=2(r+h)$$

**Figure 4:**
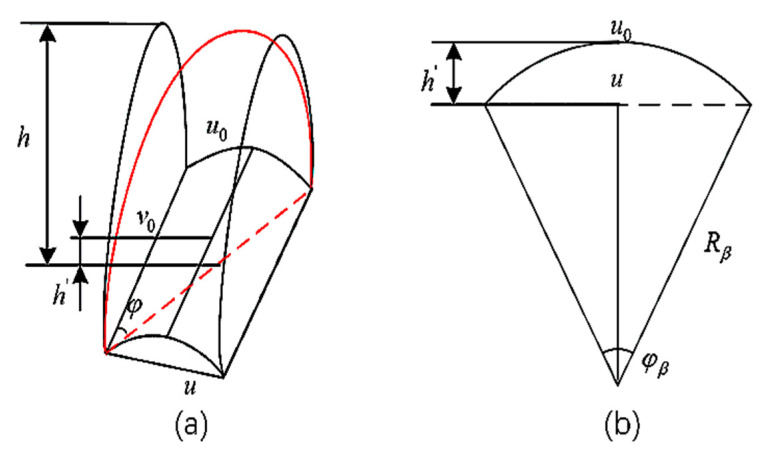
(**a**) Actuator bend segment. (**b**) Actuator bottom bend diagram.

The angle of the segment, $\varphi_{\beta }$, the radius of curvature, $R_{\beta }$, the length, *u*, the fiber helix angle, φ, and the height of the arch, $h^{\prime }$, caused by the bottom bend can be derived from the geometric relationship shown in Figure 4:

(3) $$\varphi_{\beta }=\frac{\theta }{2n}$$

(4) $$R_{\beta }=\frac{u_{0}}{\varphi_{\beta }}$$

(5) $$u=2R_{\beta }\sin\frac{\varphi_{\beta }}{2}$$

(6) $$\varphi =\arcsin\frac{u}{\sqrt{u^{2}+v_{0}^{2}}}$$

(7) $$h^{\prime }=R_{\beta }-R_{\beta }\cos\frac{\varphi_{\beta }}{2}$$

### 2.3. Preparation of Soft Actuator

**Figure 5:**
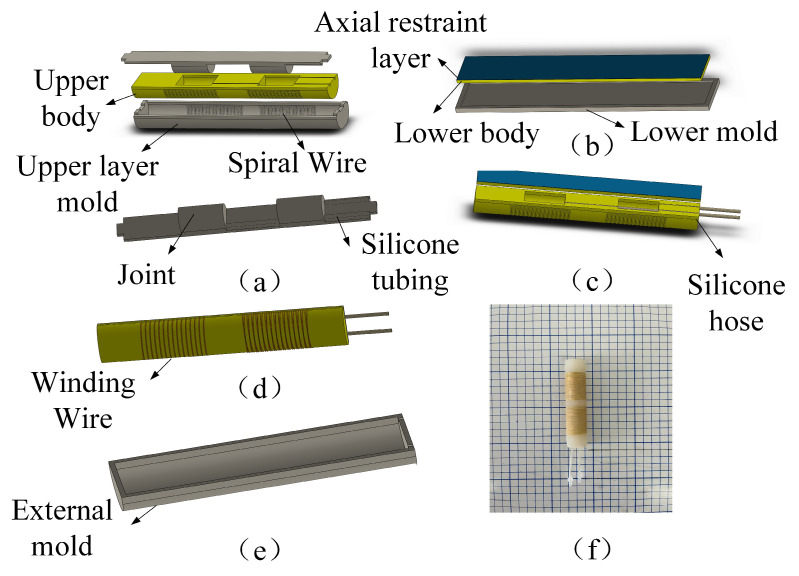
(**a**–**e**) Soft actuator production process. (**f**) Photograph of soft actuator.

Because the main part of the actuator is made of silica gel material, a casting method with a low cost and reusable mold is often adopted. Its production is mainly divided into the following steps: the production of the upper layer and the bottom layer of silica gel, bonding, and internal mold overall winding, and the production of the outer mold. Step 1: Using zero-degree silica gel, a solidifying liquid and liquid silica gel are fully mixed in a 1:1 ratio. The stirred silica gel is put into a vacuum machine to remove bubbles to reduce the influence of bubbles on the experimental results. After removing the air bubbles, the silicone is slowly poured into the inner mold (Figure 5a). At the same time, non-stretchable gauze material is placed in the bottom mold to act as the limiting layer to make the bottom layer. The upper and bottom molds with silica gel material are placed in adrying box and left to dry at 50 °C. Step 2: After demolding, the silicone hose is placed into the pipeline space reserved on the upper layer of the inner silicone mold. A layer of silicone adhesive solution is applied on the surface of the lower silicone gel to make the upper and lower layers of silicone gel bond. Step 3: The bidirectional symmetrical wire is wrapped with non-stretched nylon wire according to the spiral position in the mold to prevent the distortion of the actuator in the joint. Step 4: The inner mold is placed into the outer film mold as a whole, and then dried at the same temperature of 50 °C. The final soft actuator is shown in Figure 5f.

## 3. Experimental Platform and Performance Analysis

Since this paper adopts a flexible pneumatic actuator based on the fiber-reinforced drive principle, the flexible pneumatic actuator is optimized and physically fabricated after simulation parameters and a pneumatic control platform are identified. The bending control of the flexible actuator is realized, and the bending effect and other performances of the actuator are analyzed.

### 3.1. Soft Actuator Simulation Model and Parameter Determination

**Figure 6:**
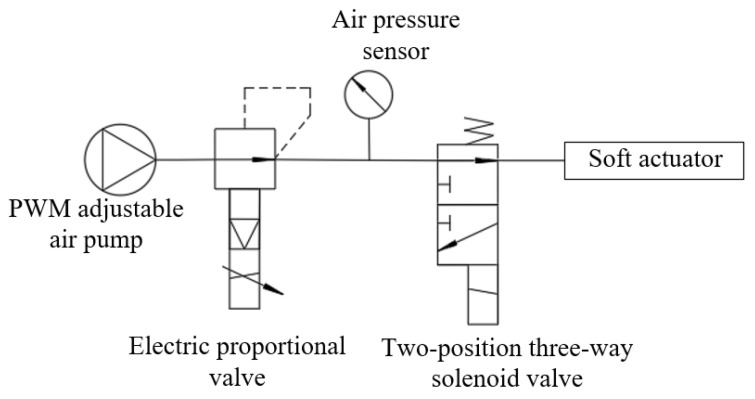
Schematic diagram of the pneumatic actuation system.

To achieve a bending effect at the joint of the soft actuator, the pneumatic execution system, as shown in Figure 6, can be used to control the gas generated by the air pump. In this paper, the Kamoer miniature vacuum air pump is selected. The maximum pressure of this air pump is 90 kPa. The size of the duty cycle of the gas pump input signal is adjusted to change the output gas flow rate, and the maximum flow rate is set to 1.1 L/min. The ITV1010-212L electrical proportional valve adjusts the gas produced by the air pump.

**Figure 7:**
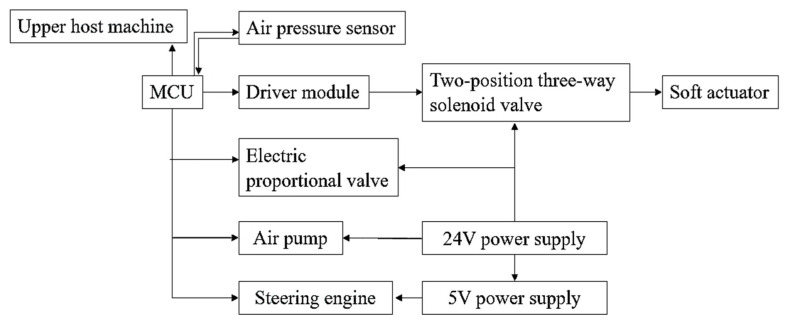
Control system flow chart.

A connected air pressure sensor can monitor the size of the air pressure value of the whole system in real time and feed it back to the host computer. After stabilizing the air pressure, the gas flows into the flexible actuator or is discharged through a two-position three-way solenoid valve to achieve the effect of inflating, exhausting, and maintaining the actuator. The control system, as shown in Figure 7, can be used for the bending and grasping experiment on the whole palm. The signal generated by the upper computer controls the MCU to issue corresponding instructions, and to command and control the driver module, electrical proportional valve, air pump, and steering gear. The drive module composed of uln2003 can be directly connected to the two-position three-way solenoid valve to control the switch of the solenoid valve, the electric proportional valve is used to determine the setting of the system pressure value, and the air pump is used to generate the gas required by the bending of the driver. The steering gear installed on the thumb can increase the degree of freedom of the thumb and thus drive the thumb to rotate at the corresponding angle on the palmar surface.

### 3.2. Soft Actuator Performance Analysis

**Figure 8:**
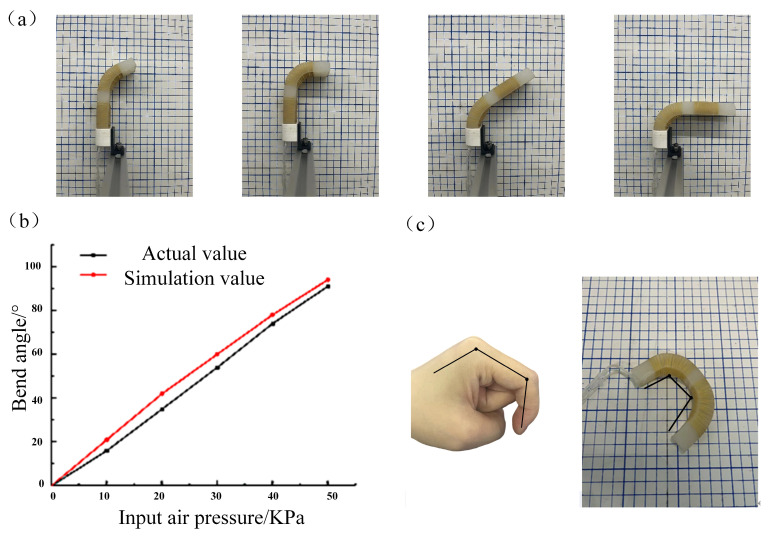
(**a**) Soft actuator joint bending effect. (**b**) Comparison of experimental and simulated bending angles. (**c**) Soft actuator bending vs. human finger bending.

The gas produced by the air pump, through the input to the electrical proportional valve, can be output within the specified range of any pressure value. The gas output size was set as 0–50 kPa, and the gas pressure was gradually increased by 10 kPa to perform a bending experiment on the two joints of the actuator. Figure 8a shows the actuator’s bending effect. The bending angle of the actuator is recorded under different pressures. Figure 8b shows the linear relationship between the actuator input pressure value and the bending angle. When the input pressure value is 50 kPa, the bending angle of the actuator can reach 91°. When an equal amount of air pressure is applied simultaneously to both joints of the soft actuator, the actuator is bent to match the bending characteristics of the human finger. The bending effect is shown in Figure 8c. The results show that the actuator can achieve a bending effect under the pressure of both joints which is very similar to the bending effect of human fingers.

**Figure 9:**
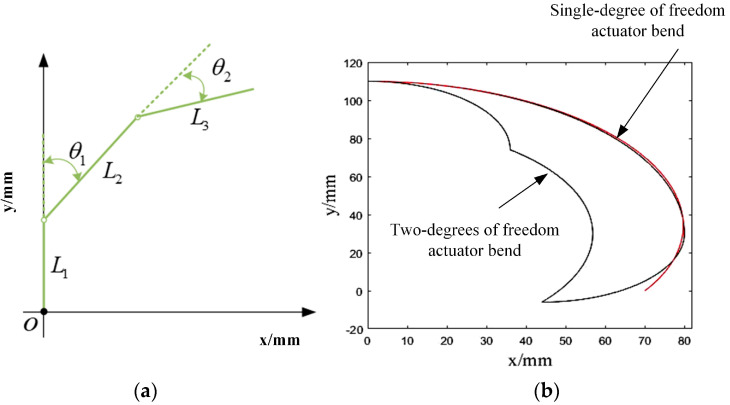
(**a**) Soft actuator structure simplification. (**b**) Comparison of single-degree-of-freedom and double-degree-of-freedom soft actuator end trajectory.

To better analyze the bending characteristics of the actuator, corresponding model simplification of the soft actuator can be carried out (Figure 9a). The designed double-joint soft actuator is simplified into a three-link mechanism with the midpoint of the actuator air cavity length as the bending point. The expression of the equation of the actuator fingertip trajectory is obtained by using the edge–angle relationship as follows:

(8) $$\begin{array}{l} x=L_{1}+L_{2}\cos\theta_{1}+L_{3}\cos(\theta_{1}+\theta_{2})\\ y=L_{2}\sin\theta_{1}+L_{3}\sin(\theta_{1}+\theta_{2}) \end{array}$$

where *L* represents the length of the connection, $L_{1}=30$ mm, $L_{2}=44$ mm, $L_{3}=36$ mm, θ represents the bending angle, and $\theta_{1}=\theta_{2}\in \left[0,\frac{\pi }{2}\right]$. By substituting in Formula (8), the motion trajectory of the actuator fingertip can be obtained. The motion trajectory is shown in Figure 9b. The fingertip movement trajectory indicates the grasping range of the soft actuator. On the premise of having the same grasping range, compared with the circular arc of the end trajectory of the traditional single-degree-of-freedom soft actuator, the designed double-joint soft actuator shows a new crescent shape when bending [29]. The new soft actuator is more adaptable to human finger characteristics and offers more flexibility when reaching the same bending range.

**Figure 10:**
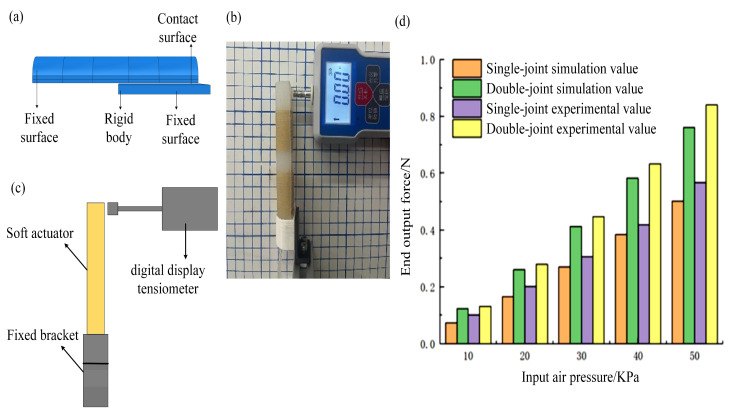
(**a**) Soft actuator end force simulation model. (**b**,**c**) Actual measurement experiment platform. (**d**) Comparison of experimental and simulated values of end force.

The measurement platform of the actuator end output force was built into the finite element simulation, as shown in Figure 10a. In the soft finite element simulation, the actuator’s bending direction is fixed to a rectangular rigid object, and the actuator contacts the object in its bending state. As the pressure in the actuator chamber increases, the actuator squeezing pressure on the rigid object increases. By calculating the resultant force generated by force on the object’s surface, the output force at the end of the actuator is obtained. When measuring the output force size at the end of a single joint, finger bone 1 should be fixed, and when calculating the double joint, the metacarpal portion of the actuator should be fixed. Both the finite element simulation and the experiment adopted the same fixed method by fixing the gas input terminal simultaneously. During the experimental measurement (as shown in Figure 10b,c), to eliminate the influence of gravitational potential energy on the results, the actuator was placed vertically, and the output force at its end was measured using the tension meter placed horizontally. The measured result of the output force at the end of the actuator is shown in Figure 10d. The test results show that when the input air pressure value is 50 kPa, the actual measured value of the end output force can reach 0.50 N and 0.84 N for single- and double-joint bending, respectively, which can meet the fingertip manipulation grasp of general objects. The experimental value of the actuator end output force is very close to the simulated value, which effectively proves the feasibility of the experiment.

**Figure 11:**
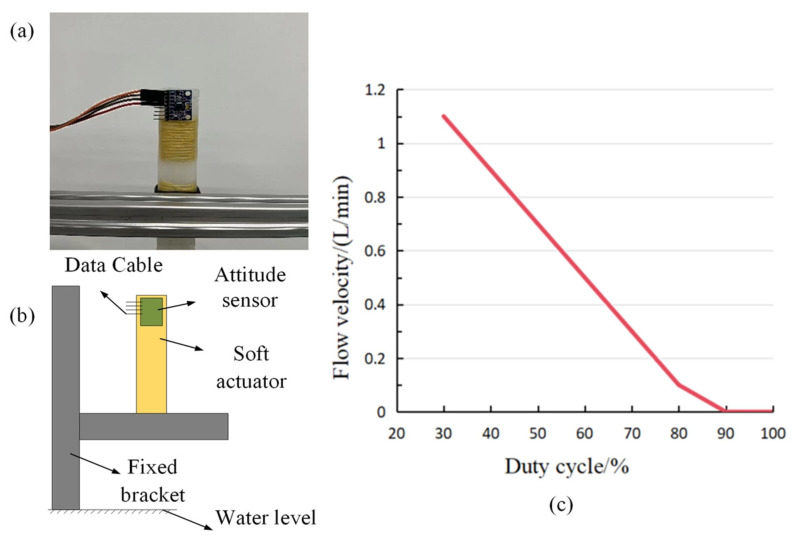
(**a**,**b**) Bending speed experimental measurement platform. (**c**) Air pump duty cycle and gas flow rate relationship.

**Figure 12:**
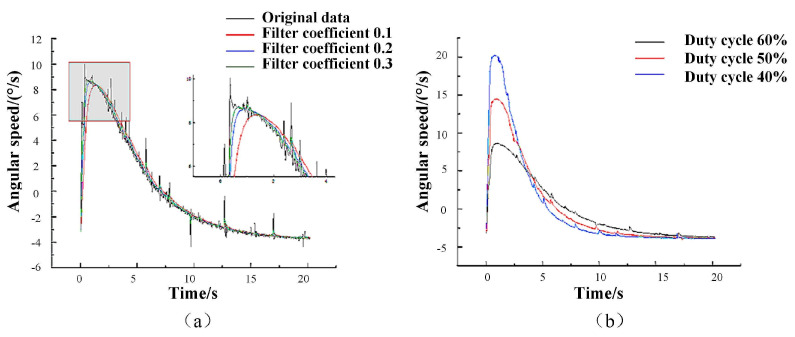
(**a**) The influence of the selection of raw data and different filtering coefficients on the results of experimental data. (**b**) The relationship between the magnitude of the duty cycle of the air pump input signal and the angular velocity.

The demand for rapid and precise actuator bending motion is prevalent in various applications, such as minimally ivasive medical surgeries or intricate assembly line tasks. To enhance the control and flexibility of our soft pneumatic actuators, it is imperative to comprehend the correlation between the air pump’s output gas flow and the actuator’s bending speed. This understanding will enable us to optimize the control algorithm of the soft pneumatic actuator, thereby achieving swifter and more accurate motion. The experimental measurement platform shown in Figure 11a,b is used to measure the relationship between the bending speed of the actuator and the output gas flow rate of the gas pump when bending. By changing the input signal size of the gas pump, the output gas flow rate of the gas pump can be changed (the relationship between gas flow rate and the input signal size is shown in Figure 11c, and it corresponds to the angular speed of the finger during bending), and thus can affect the speed of the dexterous hand grasping the object. Through placing the mpu6050 attitude sensor vertically at the end of the actuator, the relationship between the angular speed and time change when the actuator is bent is measured, and the measurement results of the three experiments are averaged. The first-order low-pass filter is used to filter the original data. The effects of the original data and the selection of different filter coefficients on the experimental data results are shown in Figure 12a. The relationship between the magnitude of the duty cycle of the gas pump input signal and the angular speed is shown in Figure 12b. The rapidity of the gas pump output gas flow has a significant effect on the rapidity of actuator bending.

**Figure 13:**
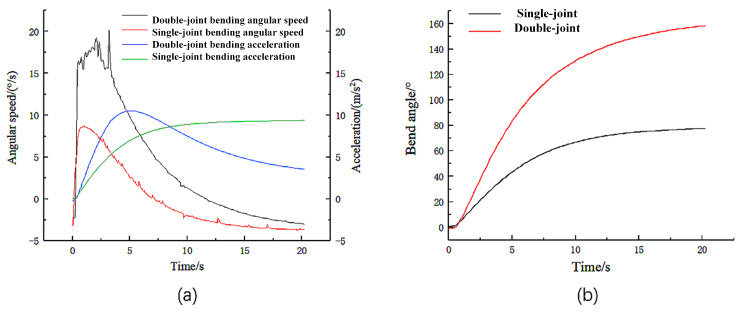
(**a**) Variation in angular velocity and acceleration with time when the duty cycle is 60%. (**b**) Relationship between angle and time.

As shown in Figure 13, the duty cycle of the air pump input signal was adjusted to 60%, and gas was introduced to the single and double joints of the actuator to observe the angular speed, acceleration, and angle change curves with time during the bending of the actuator. Due to the joint material’s softness, the actuator’s angular velocityjittered when the double joint was bent. The speed of the output gas flow from the air pump significantly influences the bending speed of the actuator. By precisely controlling the output gas speed of the air pump, we can further enhance the flexibility and accuracy of actuator movement, providing essential data support for subsequent dexterous hand production. In subsequent experiments, the input signal size of the air pump for actuator bending speed should be adjusted, while dexterous hands can enable adjustments in grasping object speed and accuracy.

## 4. Experimental Study of Dexterous Hand

An 11-degree-of-freedom dexterous hand was constructed based on a designed double-joint soft actuator, and hand grasping experiments were conducted.

### 4.1. Dexterous Hand and General Grasping

**Figure 14:**
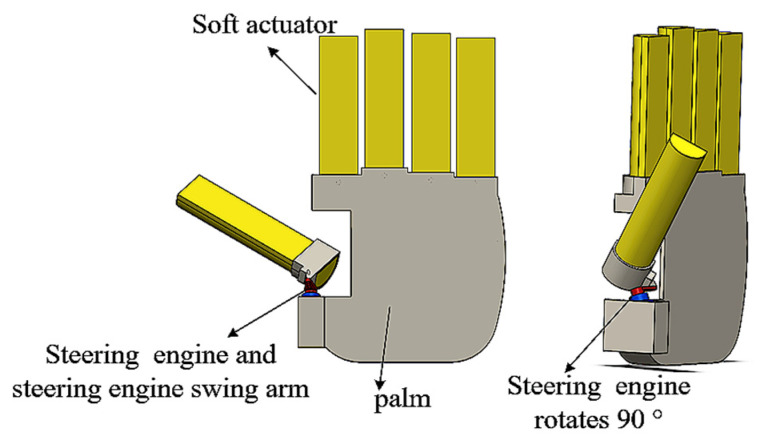
Five-finger dexterous hand structure.

**Figure 15:**
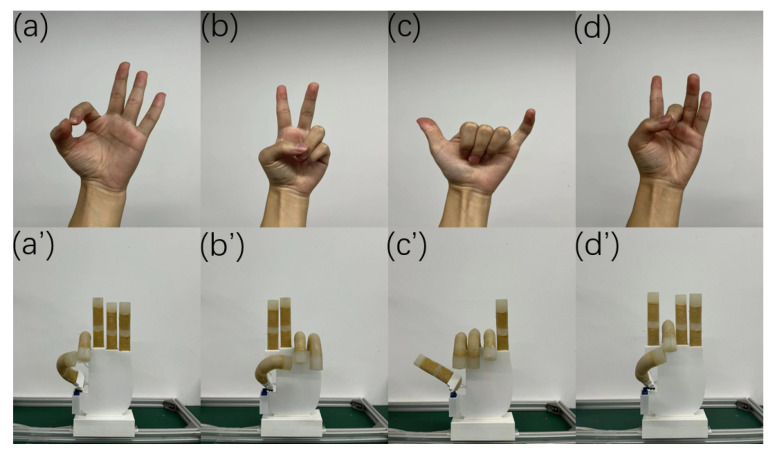
Dexterous hand; four gestures. (**a**–**d**,**a′**–**d′**) show the “Ok” gesture, the victory gesture, the “six” gesture and the orchid hand gesture, respectively.

**Figure 16:**
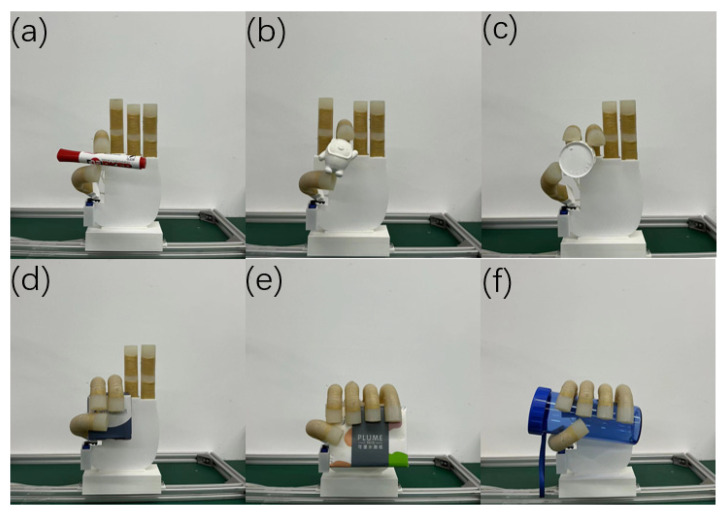
Dexterous hand grasping experiment. (**a**,**b**) Two-finger grasp. (**c**,**d**) Three-finger grasp. (**d**–**f**) Five-finger grasp.

As shown in Figure 14, a five-finger dexterous hand equipped with an actuator is introduced, in which the steering engine installed in the heel part of the thumb can drive the thumb as a whole to rotate and move on the palmar surface of the hand. The maximum rotation angle of the steering engine is set to 90°, which can achieve cooperation between the thumb and the index finger, middle finger, or ring finger to grasp. The input gas pressure size is changed, and the bending angle of the finger is adjusted; the solenoid valve’s switch can change the gas flow direction. Thus, different gestures or fits can be realized (Figure 15). Figure 16 shows that by applying the dexterous hand to the fingertip grasping or enveloping objects, the dexterous hand can achieve the stable grasping of objects of different sizes through different grasping methods. The dimensions and weight of each object are shown in Table 2.

### 4.2. Length Grip

**Figure 17:**
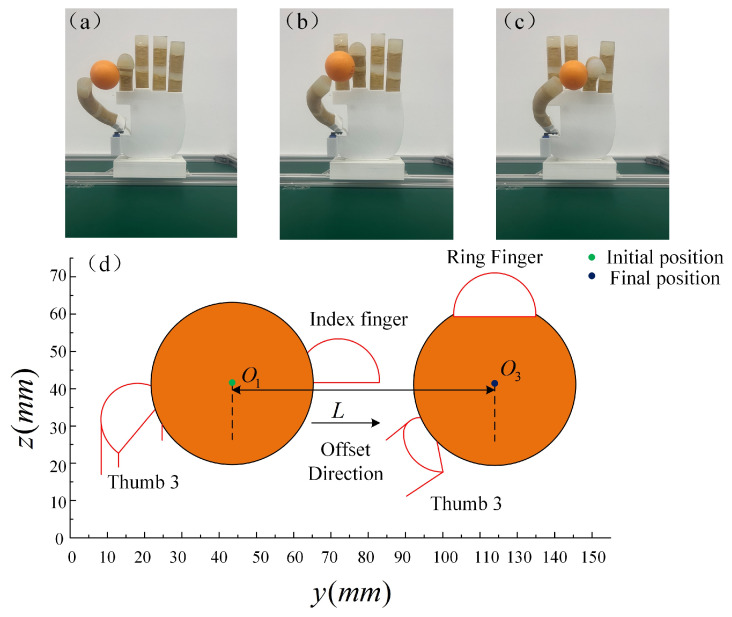
The horizontal movement experiment. (**a**–**c**) The ball moving in a horizontal direction. (**d**) Horizontal experimental model.

The horizontal movement experiment is shown in Figure 17a–c. A sphere with a diameter of 40 mm is grasped via passive adaptation using two fingertips, the input gas pressure value is set to 30 kPa, and the steering engine where the thumb is located is rotated to angles of 50°, 70°, and 90°, so that the thumb, in turn, cooperates with the index finger, middle finger, and ring finger to grasp the ball for the experiment. The experimental measurement of the ball is performed in a vertical direction to maintain the same height in the horizontal plane to move at a distance of *L* = 95 mm.

### 4.3. High Grip

**Figure 18:**
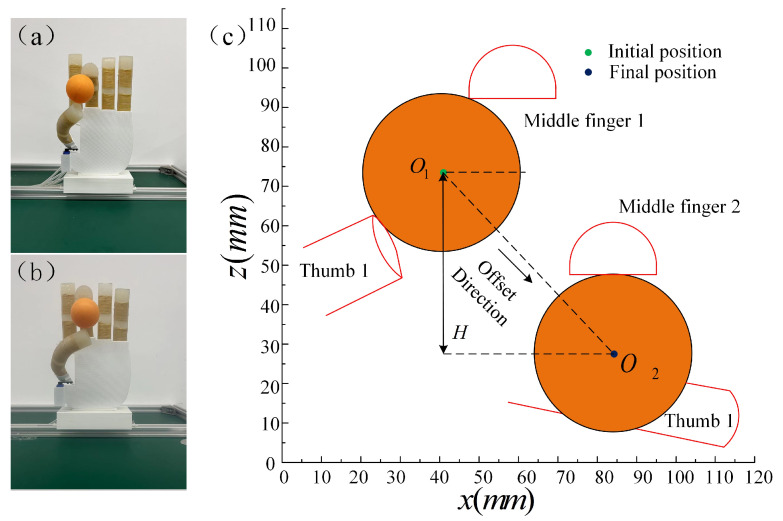
The vertical distance experiment. (**a**,**b**) The ball moves in a vertical direction. (**c**) Vertical movement model.

The vertical distance experiment is shown in Figure 18a,b, which introduces a small ball for a thumb and middle fingertip grasping experiment. During the whole grasping process, the steering gear position of the thumb is adjusted to 90°, and the air pressure size is gradually increased from the set initial value of 30 kPa to 50 kPa to observe the movement change of the small ball during the whole grasping experiment. The offset direction of the ball can be regarded as the oblique downward direction, and the height change of the ball’s center point can be regarded as the vertical direction, where H = 35 mm.

### 4.4. Angle Grabs

**Figure 19:**
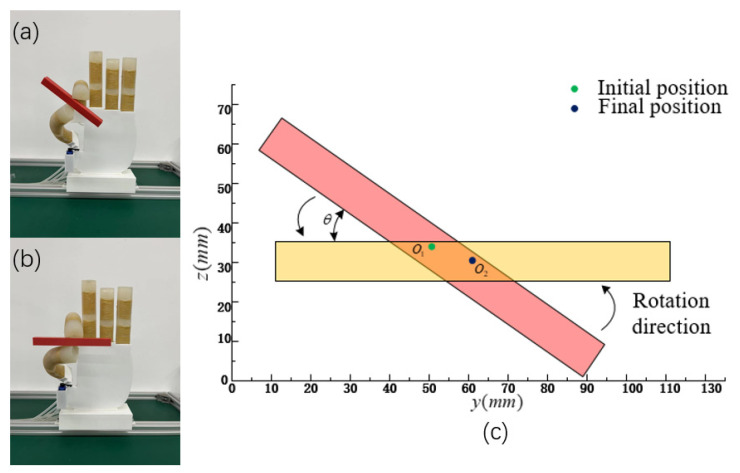
A rotation angle experiment. (**a**,**b**) Rotation angle change of rectangular objects. (**c**) Rotating experimental model.

Figure 19a,b show a small ball in a grasp in a rectangular object rotation experiment. A 3D-printed rectangular body measuring 10 × 10 × 100 mm in size is chosen, and the location of the thumb is set to 65°. The thumb and index fingertips engage in the grasping process. The initial air pressure size is set to 35 kPa, and the rectangular body is positioned at a horizontal angle. When the air pressure value size adjusted to 45 kPa, the rectangular object can be made to rotate counterclockwise to the horizontal position. In other words, by changing the air pressure by 10 kPa throughout the experiment, the rectangular object rotates 37° counterclockwise.

The experimental results show that the dexterous hand, composed of soft actuators, is highly flexible and can implement human gestures and grasping operations of objects commonly found in daily life.

## 5. Conclusions

This paper proposes a dual-degree-of-freedom soft actuator based on the fiber winding drive principle to distinguish traditional single-degree-of-freedom mechanical bending. Double-joint bending matches human finger bending and achieves a bending space similar to that of the human finger, as described via the manufacturing process, which analyzes the actuator’s performance through a single actuator experiment. We propose changing the actuator’s bending speed by adjusting the air pump’s output flow. Based on the ten degrees of freedom of five fingers, the whole dexterous hand can reach eleven degrees of freedom by adding the steering gear to the root of the thumb to add an extra degree of freedom. The feasibility and flexibility of the robot’s dexterous hands were verified through gesture recognition and grasping experiments.

We propose four grasping gestures of dexterous hands, and conducted five in-hand grasping experiments, including general object grasping, sphere grasping, and horizontal, vertical, and rotational grasping movements. The results demonstrate that the five-finger dexterous hand exhibits a wide range of graspability, strong adaptability, and a high anthropomorphism level. Furthermore, the results confirm that the five-finger dexterous hand possesses exceptional flexibility and excellent grasping capabilities, which can be highly valuable in industrial and medical applications.

Future work includes the use of specific devices to accurately identify the corresponding trajectory of the actuator during bending in order to optimize the structure of the actuator, while the gripping strength of the actuator can be improved by using harder silicone materials or adding rigid objects to the end of the actuator.

## Figures and Tables

**Table 1:** Actuator structure parameters.

Parameter of Dimension	The Numerical Value (Unit/mm)
Air chamber inner diameter, *r*	8
Rectangle width, *b*	2
Air chamber thickness, *h*	3
Bottom layer thickness, *t*	3
Joint length, *l*	20
Metacarpal	20
Finger bone 1	24
Finger bone 2	26

**Table 2:** The mass and size of the objects.

Serial Number	Designation	Quality/g	Size/mm
1	Marking pen	13.9	17 × 17 × 135
2	Bing Dwen Dwen	35.2	52 × 32 × 50
3	Bottle cap	5.6	48 × 48 × 23
4	Carton	18.4	67 × 67 × 52
5	Tissue	68.3	114 × 88 × 56
6	Cup	123.5	64 × 64 × 160

## Data Availability

The data that support the findings of this study are available from the corresponding authors upon reasonable request.
